# A Novel Non-Invasive Scoring System for Differentiating DFSP and Keloids

**DOI:** 10.1055/a-2823-0117

**Published:** 2026-06-15

**Authors:** Asako Iida, Hiroaki Kuwahara, Satoshi Akaishi, Rei Ogawa

**Affiliations:** 1 Department of Plastic and Reconstructive Surgery, Nippon Medical School Musashi Kosugi Hospital, Kanagawa, Japan; 2 Department of Plastic, Reconstructive and Aesthetic Surgery, Nippon Medical School Hospital, Tokyo, Japan

**Keywords:** DFSP, keloids, differential diagnosis method

## Abstract

**Background:**

Differentiating dermatofibrosarcoma protuberans (DFSP) from keloids presents significant clinical challenges, particularly when skin biopsies are contraindicated. This study introduces a novel, non-invasive diagnostic method leveraging the Japan Scar Workshop Scar Scale (JSS) combined with the cosine of the angle (cosθ) to distinguish between these conditions.

**Methods:**

In this case–control study, 43 cases were retrospectively analyzed using the new evaluation method, JSS × cosθ, designed specifically for this differential diagnosis. This method applies JSS in conjunction with cosθ to assess lesion characteristics non-invasively.

**Results:**

The JSS × cosθ scores effectively differentiated between DFSP and keloids. Scores ≥16 were indicative of keloids, whereas scores <16 suggested DFSP, with these findings achieving statistical significance ( *p* < 0.001).

**Conclusion:**

The JSS × cosθ scoring system provides a reliable and safe diagnostic tool for distinguishing DFSP from keloids, particularly in cases where biopsy is avoided due to the risk that stimulation of the reticular dermis may lead to the development, enlargement, or worsening of keloids. This method promises to significantly impact the specific management of DFSP and keloids.

## Introduction

Dermatofibrosarcoma protuberans (DFSP), first named by Hoffman in 1925, is a rare intermediate-grade soft tissue tumor associated with the COLIA1-PDGFB fusion protein resulting from a t(17;22) chromosomal translocation. 1 DFSP typically arises in the dermis, infiltrates the subcutaneous tissues, and predominantly affects the trunk, extremities, and head and neck regions. It accounts for approximately 0.1% of all malignant tumors and 1.8% to 6.0% of soft tissue malignancies, with a higher incidence in adults aged 20 to 50 years. Initially presenting as red or purple skin pigmentation, DFSP enlarges slowly to form nodules. Although the local recurrence rate is generally high, the risk of metastasis is low, with the lungs being the most common site of metastasis. However, the risk of metastasis increases to approximately 5% when the tumor undergoes a fibrosarcomatous transformation. 1 Diagnosis is confirmed by histopathological examination, which shows characteristic patterns such as storiform or cartwheel arrangements of tumor cells, with minimal cellular atypia and honeycomb infiltration into the subcutaneous adipose tissue. Immunohistochemical staining typically shows CD34 positivity. 2 Standard treatment involves wide local excision with recommended margins of 2 to 3 cm to reduce the risk of recurrence. 3 In cases where surgical intervention is challenging or metastasis is present, treatment options include radiation therapy or chemotherapy, including molecular-targeted therapies such as tyrosine kinase inhibitors. 4

Keloids are inflammatory raised lesions characterized by collagen overproduction triggered by chronic stimulation of the reticular layer of the dermis. 5 These lesions often extend beyond the original wound boundaries into the surrounding skin, leading to clinical challenges in differentiating keloids from benign or malignant conditions. At our specialized keloid clinic, cases initially diagnosed and treated with repeated steroid injections that are later identified as DFSP are frequently encountered. Keloids and DFSP are fibrogenic disorders that originate in the dermis and share a similar appearance. 1 5 Additionally, stimulation of the reticular dermis can lead to the development, enlargement, and exacerbation of keloids; therefore, skin biopsy is generally avoided in such cases. This avoidance may contribute to misdiagnosis and inappropriate treatment. A delayed diagnosis of DFSP can result in tumor enlargement, treatment complications, distant metastasis, and sarcomatous transformation, necessitating early diagnosis and treatment. 1

Previous studies have shown that keloids tend to expand with skin tension. 6 This characteristic could be used to develop a novel evaluation method for differentiating DFSP from keloids. The significance of this study lies in its provision of an early and accurate differential diagnosis between keloid and DFSP to optimize treatment plans and improve treatment outcomes. Especially when skin biopsy tends to be avoided for keloids, there is a need for a safe and non-invasive differential diagnostic method. This study aimed to verify the efficacy of a new evaluation method using the Japan Scar Workshop Scar Scale (JSS) 7 combined with cosθ (JSS × cosθ) for differentiating between DFSP and keloids.

## Methods

**Fig. 1:**
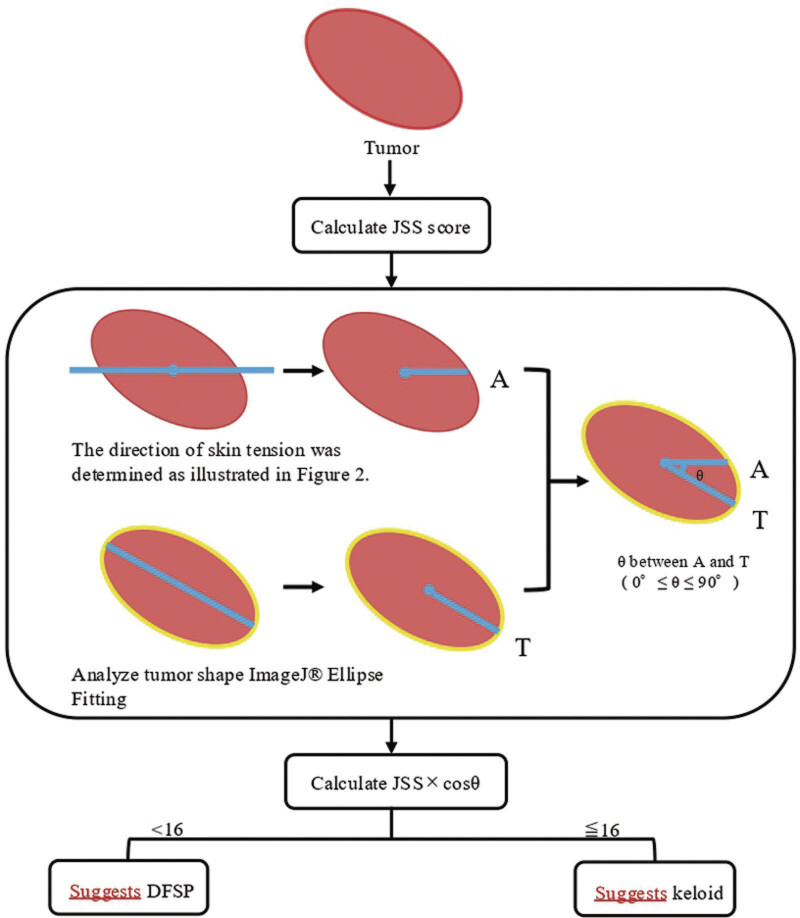
Flowchart of case selection criteria identified through PubMed search.

In this case–control study, four of our own cases, along with 39 cases identified through a PubMed search conducted on July 20, 2024, were reviewed, and cases were selected from the most relevant articles. This study was limited to the trunk because the distribution of tension in the limbs is complex. We excluded cases in which the tumor margins were indistinct, making it difficult to accurately assess the direction of skin tension, or when the lesion was too irregular in shape to measure the long axis consistently. Tumors that lacked sufficient imaging detail to determine these factors and those with ambiguous pathological diagnoses were also excluded. As a result, 39 cases from PubMed were included in the analysis. This approach helped minimize selection bias while ensuring the inclusion of diagnostically valuable cases ( Fig. 1 ).

**Fig. 2:**
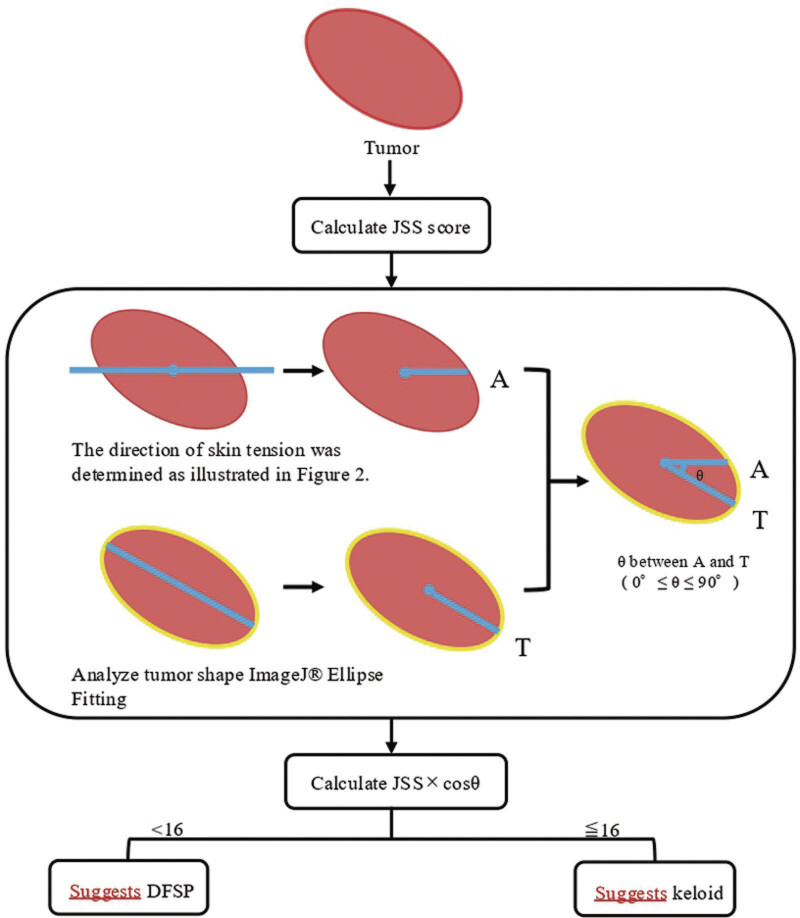
Illustration showing the direction of skin tension.

**Fig. 3:**
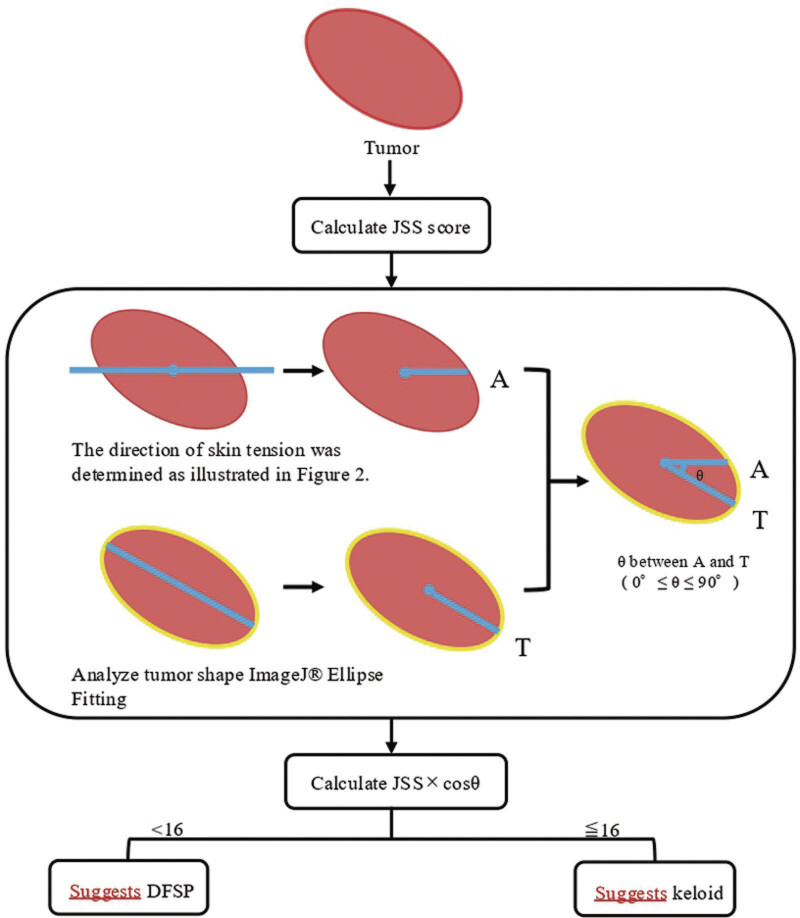
DFSP case, diagram depicting the measurement method for JSS × cosθ, for differentiating DFSP from keloids. DFSP, dermatofibrosarcoma protuberans; JSS, Japan Scar Workshop Scar Scale.

First, each tumor was scored using the JSS, which is typically used for diagnosing keloids (0–5: matured scars, 6–15: hypertrophic scars, 16–25: keloids; Table 1 ). 7 The shape of each tumor was then analyzed using the Ellipse Fitting tool in ImageJ® software (National Institutes of Health, Bethesda, MD), 8 determining the major axis A. The keloid expansion direction T was identified based on the skin tension direction ( Fig. 2 ). According to the report by Akaishi et al, the direction of keloid expansion tends to align with the direction of skin tension 1, which often approximates a direction perpendicular to the relaxed skin tension lines. 6 The angle θ between A and T (0 degrees ≤ θ ≤90 degrees) was measured ( Figs. 3 4 5 ). The JSS × cosθ product was calculated as the new evaluation method, and the scores for each tumor were determined ( Supplementary Table S1 [available in the online version only]). In this study, the cosine value was introduced to minimize the influence of alignment between the lesion's major axis and the direction of skin tension when differentiating between keloids and DFSP. The scores were compared with the established keloid diagnostic value (JSS, 16 points) using a one-sample *t* -test to assess statistical significance. Statistical significance was set at *p* < 0.05. Statistical analyses were performed using SPSS (version 22.0; IBM Japan, Tokyo, Japan). This study was approved by the Institutional Review Board of our institution (approval no. 763-5-73). Written informed consent was obtained from the patient for the publication of their photographs and relevant medical information in this study, while data for the remaining cases were derived from previously published studies.

**Fig. 4:**
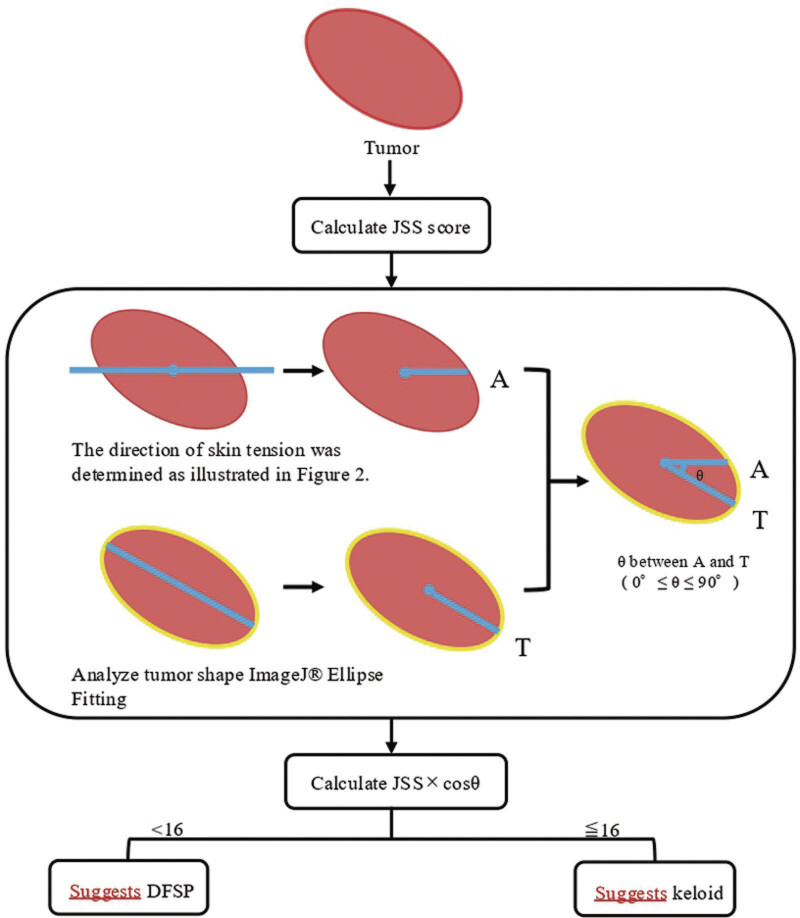
Keloid case, diagram depicting the measurement method for JSS × cosθ, for differentiating DFSP from keloids. DFSP, dermatofibrosarcoma protuberans; JSS, Japan Scar Workshop Scar Scale.

**Fig. 5:**
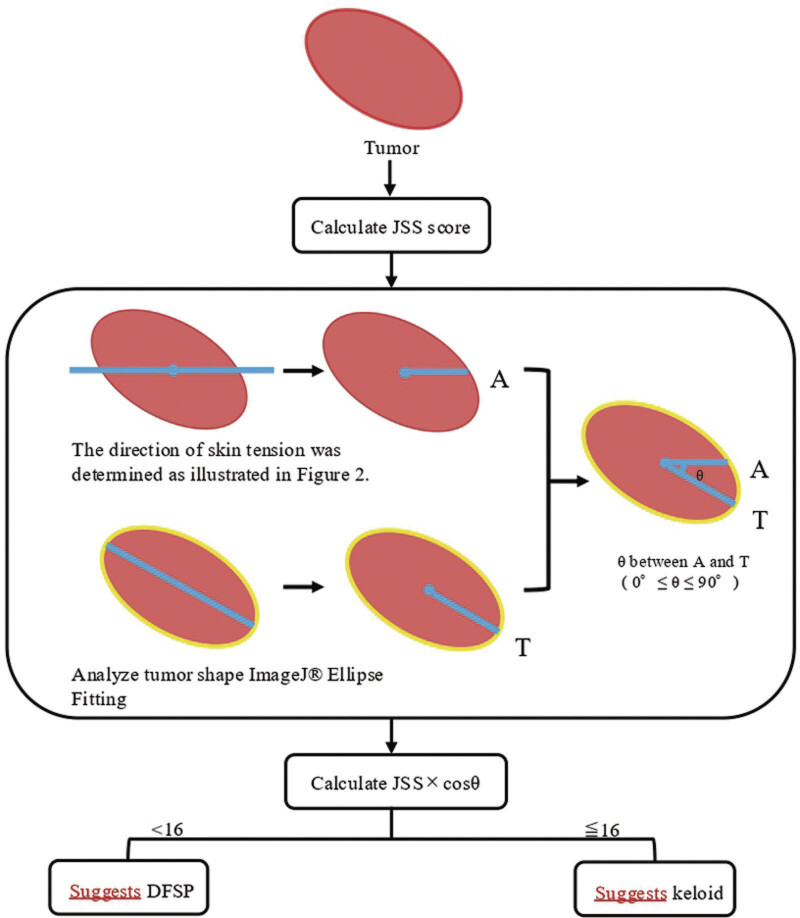
Diagnostic process for differentiating DFSP from keloids. DFSP, dermatofibrosarcoma protuberans; JSS, Japan Scar Workshop Scar Scale.

**Table 1:** Keloid and hypertrophic scar classification and evaluation table 7

Classification (for grading and selection of appropriate treatment methods)				
Risk factors				
Classification factor	Condition 1	Score	Condition 2	Score
Human race	Africans Others	2 1	Caucasians	0
Familial tendency	Clearly exists	1	Not clear	0
Number	Multiple	2	Solitary	0
Region	Anterior chest, scapular–shoulder, suprapubic	2	Others	0
Age at onset (years)	0–30 31–60	2 1	≥61	0
Causes	Unknown or minute	3	Specific wound type	0
Current symptoms				
Symptom	Condition 1	Score	Condition 2	Score
Size	Over 20 cm ^2^	1	Under 20 cm ^2^	0
Vertical growth	Clearly exist	2	Not clear	0
Horizontal growth	Clearly exist	2	Not clear	0
Shape	Characteristic shape	3	Others	0
Erythema around scars	Clearly present	1	Not clear	0
Subjective symptom	Always exist Intermittent	2 1	None	0

Scoring method from the Japan Scar Workshop Scar Scale (JSS). 7

Scores

0–5: Normal mature scar.

5–15: Hypertrophic scar characteristics.

15–25: Keloid characteristics.

This table classifies keloids and hypertrophic scars, assigning scores based on risk factors and symptoms to select appropriate treatment methods.

## Results

The JSS × cosθ scores were <16 in 38 of 43 cases (mean score: 8.29, standard deviation: 5.69). The 95% confidence interval (CI) for the mean ranged from 6.54 to 10.04, with a minimum score of 0.00 and a maximum score of 17.12. Additionally, the 95% CI for the distribution of JSS × cosθ was 0 to 19.44. The one-sample *t* -test results indicated that the mean JSS × cosθ score was significantly lower than the established keloid diagnostic value (16 points; *p* < 0.001). In contrast, the JSS score alone did not differ significantly from this threshold ( Table 2 ). In this study, 37% of cases were diagnosed as keloids using the JSS score alone. However, by incorporating cosθ into the JSS score, the percentage of cases diagnosed as DFSP increased significantly to 88%.

**Table 2:** Statistical analysis of JSS × cosθ and Japan Scar Workshop Scar Scale in dermatofibrosarcoma protuberans cases ( *n* = 43)

	JSS × cosθ	JSS
Mean	8.29	15.77
Standard deviation	5.69	1.66
95% Confidence interval of the mean		
Upper limit	6.54	15.26
Lower limit	10.04	16.28
Minimum value	0.00	11.00
Maximum value	17.12	20.00
95% Confidence interval of distribution		
Upper limit	−2.86	12.51
Lower limit	19.44	19.02
*p* -Value (one-sample *t* -test; test value = 16)	**<0.001**	0.363

Abbreviation: JSS, Japan Scar Workshop Scar Scale.

## Discussion

This study utilized the JSS to score each tumor and verified the efficacy of a new JSS × cosθ evaluation method based on the characteristic expansion of keloids along skin tension lines. The results showed that in 38 of 43 cases, the JSS × cosθ scores were <16, with a mean score of 8.29. The JSS × cosθ scores were significantly lower than the established keloid diagnostic value of 16 points ( *p* < 0.001). The relatively narrow 95% CI for the mean score and a standard deviation of 5.69 support the reliability of this new evaluation method. Consequently, we propose the use of JSS × cosθ scores of <16 as a novel evaluation method for differentiating between DFSP and keloids. This demonstrates that incorporating cosθ into the evaluation improves the precision and accuracy of distinguishing between DFSP and keloids, particularly in cases where traditional methods may result in ambiguous diagnoses. This study demonstrates that keloids and DFSP can be distinguished with a high degree of accuracy. Given that biopsies are generally avoided in cases suspected to be keloids—potentially delaying the diagnosis of malignant conditions—early and accurate differentiation is considered clinically important.

Keloids are fibrogenic disorders caused by chronic inflammation of the reticular dermis, predominantly occurring in high-tension areas such as the anterior chest, shoulders, and pubic region. 6 Keloids expand along the lines of skin tension. Treatment primarily aims to suppress chronic inflammation and includes local steroid injections (triamcinolone acetonide), application of steroid tapes (fludroxycortide), and oral antiallergic drugs (tranilast). Surgical excision and radiation therapy are considered when improvement is insufficient. 8 Owing to their similar appearance, distinguishing between DFSP and keloids can be challenging; however, treatment approaches differ significantly. Although DFSP proliferates slowly, a delayed diagnosis can lead to tumor enlargement, treatment complications, distant metastasis, and sarcomatous transformation, necessitating early diagnosis and treatment. In contrast, keloids are generally managed with conservative therapy centered on steroid treatment, with the diagnosis based on clinical presentation due to the tendency to avoid skin biopsy.

Several other conditions that resemble DFSP and keloids, including mixed tumors of the skin, chondroid syringoma, cutaneous squamous cell carcinoma, juvenile xanthogranuloma, pseudolymphoma, nodular scleroderma, and lobomycosis, 4 should be considered in future studies.

This study has several limitations. The sample size of 43 patients is relatively small, and diagnosis may be particularly challenging when the long axis of the DFSP coincides with the direction of skin tension. Although the standard deviation was relatively low, the minimum and maximum values suggested potential variability in the application of this evaluation method across different cases, indicating the need for further validation and refinement. Moreover, because the dataset relied primarily on published images, it may have overrepresented typical, well-demarcated lesions, which could exaggerate the apparent diagnostic clarity. Skin biopsy tends to be avoided in cases of suspected keloids, even in atypical cases or when differential diagnosis with other fibrotic diseases is required. Therefore, this method should be regarded solely as an adjunct diagnostic tool and not as a substitute for histopathological confirmation. Its use should be limited to supporting the decision-making process regarding the need for a biopsy.

This study focuses on the visual differentiation of DFSP. However, considering the disease characteristics and clinical course of DFSP, we believe that 3D evaluation may also be necessary, and we would like to explore this approach in future studies. We also believe that this approach can be applied to other keloid-like diseases. We intend to accumulate cases and conduct research in the future. When keloids are clinically diagnosed or suspected, no biopsy, magnetic resonance imaging (MRI), computed tomography (CT), or ultrasound is performed. Since keloids are a constitution-based inflammatory disease, medical resources are generally not allocated to the aforementioned tests. We hope that this methodology will make it possible to rule out DFSP on visual examination in cases of suspected keloids.

In conclusion, this study proposed a novel evaluation method for differentiating DFSP from keloids and demonstrated its efficacy. The results suggest that this method could support the implementation of skin biopsy in cases in which it tends to be avoided for keloids, contributing only to the differential diagnosis of single cases of DFSP and keloids. However, owing to the limitations of this study, further large-scale research and evaluation of this method for other fibrogenic disorders are necessary. Ongoing refinement and clinical application studies are essential for the continued development of this evaluation method.

## References

[1] Allen A Ahn C Sangüeza O P Dermatofibrosarcoma protuberans Dermatol Clin 2019 37 04 483 488 31466588 10.1016/j.det.2019.05.006

[2] Vitiello G A Lee A Y Berman R S Dermatofibrosarcoma protuberans: What is this? Surg Clin North Am 2022 102 04 657 665 35952694 10.1016/j.suc.2022.05.004

[3] Harati K Lange K Goertz O A single-institutional review of 68 patients with dermatofibrosarcoma protuberans: Wide re-excision after inadequate previous surgery results in a high rate of local control World J Surg Oncol 2017 15 01 5 28056985 10.1186/s12957-016-1075-2 PMC5217543

[4] Henry O S Platoff R Cerniglia K S Tyrosine kinase inhibitors versus radiation therapy in unresectable dermatofibrosarcoma protuberans (DFSP): A narrative systematic review Am J Surg 2023 225 02 268 274 36184329 10.1016/j.amjsurg.2022.09.040

[5] Ogawa R Keloid and hypertrophic scars are the result of chronic inflammation in the reticular dermis Int J Mol Sci 2017 18 03 606 28287424 10.3390/ijms18030606 PMC5372622

[6] Akaishi S Akimoto M Ogawa R Hyakusoku H The relationship between keloid growth pattern and stretching tension: Visual analysis using the finite element method Ann Plast Surg 2008 60 04 445 451 18362577 10.1097/SAP.0b013e3181238dd7

[7] Ogawa R Akita S Akaishi S Diagnosis and treatment of keloids and hypertrophic scars-Japan Scar Workshop Consensus Document 2018 Burns Trauma 2019 7 39 31890718 10.1186/s41038-019-0175-y PMC6933735

[8] Walsh L A Wu E Pontes D Keloid treatments: An evidence-based systematic review of recent advances Syst Rev 2023 12 01 42 36918908 10.1186/s13643-023-02192-7 PMC10012475

