# Supplementary material for: A Novel Non-Invasive Scoring System for Differentiating DFSP and Keloids
Source: Arch Plast Surg. 2026 Jun 15;53(4):315–20. doi: 10.1055/a-2823-0117 (PMC13461262; doi:10.1055/a-2823-0117)
Supplement: Supplementary file 1 [file 10-1055-a-2823-0117_29351461.pdf]

case	1. Human race	2. Familial tendency	3. Number	4. Region	5. Age at onset	6. Causes	7. Size (cm2)	8. Vertical growth (elevation)	9. Horizontal growth	10. Shape	11. Erythema around scars	12. Subjective symptoms	total(JSS)	θ	JSS*cosθ
1	1	0	0	2	1	3	1	2	3	3	2	0	18	81	2.815820371
2	0	0	0	0	2	3	1	2	3	3	2	0	16	22	14.83494167
3	2	0	0	2	1	3	1	2	3	3	0	0	17	5	16.93530987
4	2	0	0	0	2	3	1	2	3	3	0	0	16	88	0.558391947
5	1	0	0	0	1	3	1	2	3	3	2	0	16	87	0.8373753
6	1	0	0	2	1	3	1	2	3	3	2	0	18	71	5.86022678
7	1	0	0	0	1	3	1	2	3	3	2	0	16	78	3.326587053
8	0	0	0	0	0	3	0	2	3	3	0	0	11	34	9.119413298
9	1	0	2	0	2	3	0	2	3	3	2	0	18	90	1.10218E-15
10	1	0	2	2	1	3	0	2	3	3	2	1	20	43	14.62707403
11	1	0	0	0	1	3	0	2	3	3	2	0	15	81	2.346516976
12	1	0	0	0	1	0	0	2	3	3	2	0	12	66	4.880839717
13	1	0	0	0	2	3	0	2	3	3	2	0	16	67	6.251698056
14	0	0	0	0	1	3	0	2	3	3	2	0	14	76	3.386906538
15	1	0	0	2	1	3	1	2	3	3	2	0	18	18	17.11901729
16	1	0	0	0	1	3	1	2	3	3	2	0	16	74	4.410197693
17	1	0	0	0	1	3	1	2	3	3	2	0	16	44	11.50943681
18	0	0	0	2	1	3	0	2	3	3	2	0	16	0	16
19	1	0	0	2	1	3	1	2	3	3	2	0	18	42	13.37660686
20	0	0	0	2	1	3	1	2	3	3	2	0	17	14	16.49502735
21	1	0	0	0	2	3	0	2	3	3	2	2	18	27	16.03811744
22	0	0	0	0	2	3	0	2	3	3	2	0	15	2	14.99086241
23	0	0	0	0	0	3	1	2	3	3	2	0	14	86	0.976590632
24	1	0	0	0	1	3	0	2	3	3	2	0	15	75	3.882285677
25	1	0	0	0	1	3	0	2	3	3	2	0	15	45	10.60660172
26	1	0	0	0	2	3	0	2	3	3	2	0	16	69	5.733887193
27	1	0	0	0	2	3	1	2	3	3	2	0	17	71	5.534658626
28	1	0	0	0	0	3	0	2	3	3	2	2	16	69	5.733887193
29	1	0	0	0	2	3	0	2	3	3	2	0	16	83	1.949909494
30	1	0	0	0	2	0	0	2	3	3	2	0	13	15	12.55703574
31	0	0	0	0	2	3	1	2	3	3	2	0	16	87	0.8373753
32	0	0	0	0	2	3	1	2	3	3	2	0	16	35	13.10643271
33	0	0	0	0	1	3	0	2	3	3	2	0	14	19	13.23726006
34	0	0	0	0	2	3	0	2	3	3	2	0	15	59	7.725571124
35	1	0	0	0	1	3	0	2	3	3	2	0	15	86	1.046347106
36	1	0	0	2	2	0	1	2	3	3	2	0	16	12	15.65036161
37	1	0	0	0	2	3	0	2	3	3	2	0	16	89	0.279238503
38	1	0	0	0	1	3	1	2	3	3	2	0	16	43	11.70165923
39	1	0	0	0	1	3	0	2	3	3	2	0	15	57	8.169585525
40	1	0	0	0	2	3	1	2	3	3	2	0	17	35	13.92558475
41	1	0	0	0	2	3	1	2	3	3	2	0	17	21	15.87086725
42	1	0	0	0	2	3	1	2	3	0	2	0	14	69	5.017151294
43	1	0	0	0	1	0	1	2	3	3	3	0	14	50	8.999026536
